# A chromosome-level genome assembly of the common eider, *Somateria mollissima* (Linnaeus, 1758)

**DOI:** 10.1093/jhered/esae042

**Published:** 2024-09-04

**Authors:** Ole K Tørresen, Oliver Kersten, Benedicte Garmann-Aarhus, Morten Helberg, Ave Tooming-Klunderud, Morten Skage, Sanne Boessenkool, Kjetill S Jakobsen

**Affiliations:** Department of Biosciences, Centre for Ecological and Evolutionary Synthesis, University of Oslo, Oslo, Norway; Department of Biosciences, Centre for Ecological and Evolutionary Synthesis, University of Oslo, Oslo, Norway; Natural History Museum, University of Oslo, Oslo, Norway; BirdLife Norway, Sandgata 30 B, 7012 Trondheim, Norway; Department of Biosciences, Centre for Ecological and Evolutionary Synthesis, University of Oslo, Oslo, Norway; Department of Biosciences, Centre for Ecological and Evolutionary Synthesis, University of Oslo, Oslo, Norway; Department of Biosciences, Centre for Ecological and Evolutionary Synthesis, University of Oslo, Oslo, Norway; Department of Biosciences, Centre for Ecological and Evolutionary Synthesis, University of Oslo, Oslo, Norway

**Keywords:** arctic, aves, duck, Earth Biogenome Project Norway, haplotype-resolved

## Abstract

The common eider, *Somateria mollissima mollissima* (Chordata; Aves; Anseriformes; Anatidae), is a large sea duck with a circumpolar distribution. We here describe a chromosome-level genome assembly from an individual female. The haplotype-resolved assembly contains one pseudo-haplotype spanning 1,205 megabases (with both Z and W sex chromosomes) and one pseudo-haplotype spanning 1,080 megabases. Most of these two assemblies (91.13% and 93.18%, respectively) are scaffolded into 32 autosomal chromosomal pseudomolecules plus Z and W for pseudo-haplotype one. The BUSCO completeness scores are 94.0% and 89.9%, respectively, and gene annotations of the assemblies identified 17,479 and 16,315 protein coding genes. Annotation of repetitive sequences classify 17.84% and 14.62% of pseudo-haplotype one and two, respectively, as repeats. The genome of the common eider will be a useful resource for the widely distributed northern species in light of climate change and anthropogenic threats.

## Introduction

The common eider (*Somateria mollissima*, Linnaeus, 1758, hereafter “eider”) is the largest and heaviest duck in the northern hemisphere and has a circumpolar distribution across the Arctic (Waltho and Coulson 2015; BirdLife International 2018). Eiders breed in colonies on islands and spits usually along low-lying rocky marine coasts and estuaries. They disperse along shallow seashores in winter during which they are commonly found in bays and river mouths. The female eider, which exhibits strong natal philopatry and usually returns to the same breeding colony, has a cryptic coloration with warm brown and barred black (Waltho and Coulson 2015; BirdLife International 2018). The male eider, on the other hand, has an iconic outer appearance with a black coronal region, white head, neck, and throat with pale green patches on the nape, white upper breast and heavily suffused cream to pinkish buff (Waltho and Coulson 2015; BirdLife International 2018). Apart from their ecological importance, such as enriching the nutrient levels of the aquatic and terrestrial ecosystems through the deposition of large amounts of droppings (Waltho and Coulson 2015; Clyde et al. 2021), eiders are known for their thick and warm down. Historically, these down feathers that the female plucks from her own breast and uses to line her nest, have been harvested for filling pillows and quilts and are still a highly sought-after resource as insulating material today (Snæbjörnsson -; Bédard et al. 2008).

Eiders are currently divided into six subspecies, which include *S. m. mollissima* (from northwestern Europe to Novaya Zemlya), *S. m. borealis* (from Franz Josef Land and Svalbard, across the Arctic, to Baffin Island; also Greenland and Iceland), *S. m. faeroeensi* (Faroe Islands), *S. m. dresseri* (northeastern USA and southeastern Canada), *S. m. sedentaria* (almost exclusively in Hudson and James Bay), and *S. m. v-nigrum* (from northeast Siberia to northwest North America) (Waltho and Coulson 2015; BirdLife International 2018). Subspecies differ morphologically in the male bill morphology, as well as plumage patterns of males and females (Furness et al. 2010; Waltho and Coulson 2015). Although there is evidential support for the existence of six subspecies by microsatellite and mitochondrial DNA data, genetic differentiation in microsatellite loci at the population level is weak (Furness et al. 2010; Sonsthagen et al. 2011; Waltho and Coulson 2015). Moreover, discordance between genetic and morphological assignment has been observed across several areas of the distribution possibly driven by differences in post-glacial colonization history or other ecological factors, such as overwintering grounds (Furness et al. 2010; Sonsthagen et al. 2011; Waltho and Coulson 2015). Disentangling population structure and gene flow in vagile seabirds require powerful genome-wide analyses (Kersten et al. 2021). Given the rapidly changing Arctic environment and varying population trends of eider colonies over the past decades (BirdLife International 2018; Kersten et al. 2021; Noel et al. 2021) such analyses are overdue. Genome-wide population studies strongly benefit from access to a reference genome. Here, we present a haplotype-resolved assembly of the common eider (*S. m. mollissima*) genome generated using PacBio HiFi long-read and Hi-C sequencing data. The public availability of this reference genome will facilitate further genomic research on genetic diversity, population structure, and local adaptation of the eider. This haplotype-resolved genome assembly is generated as part of the Earth Biogenome Project Norway.

## Methods

### Sample acquisition and DNA extraction

**Fig. 1.**
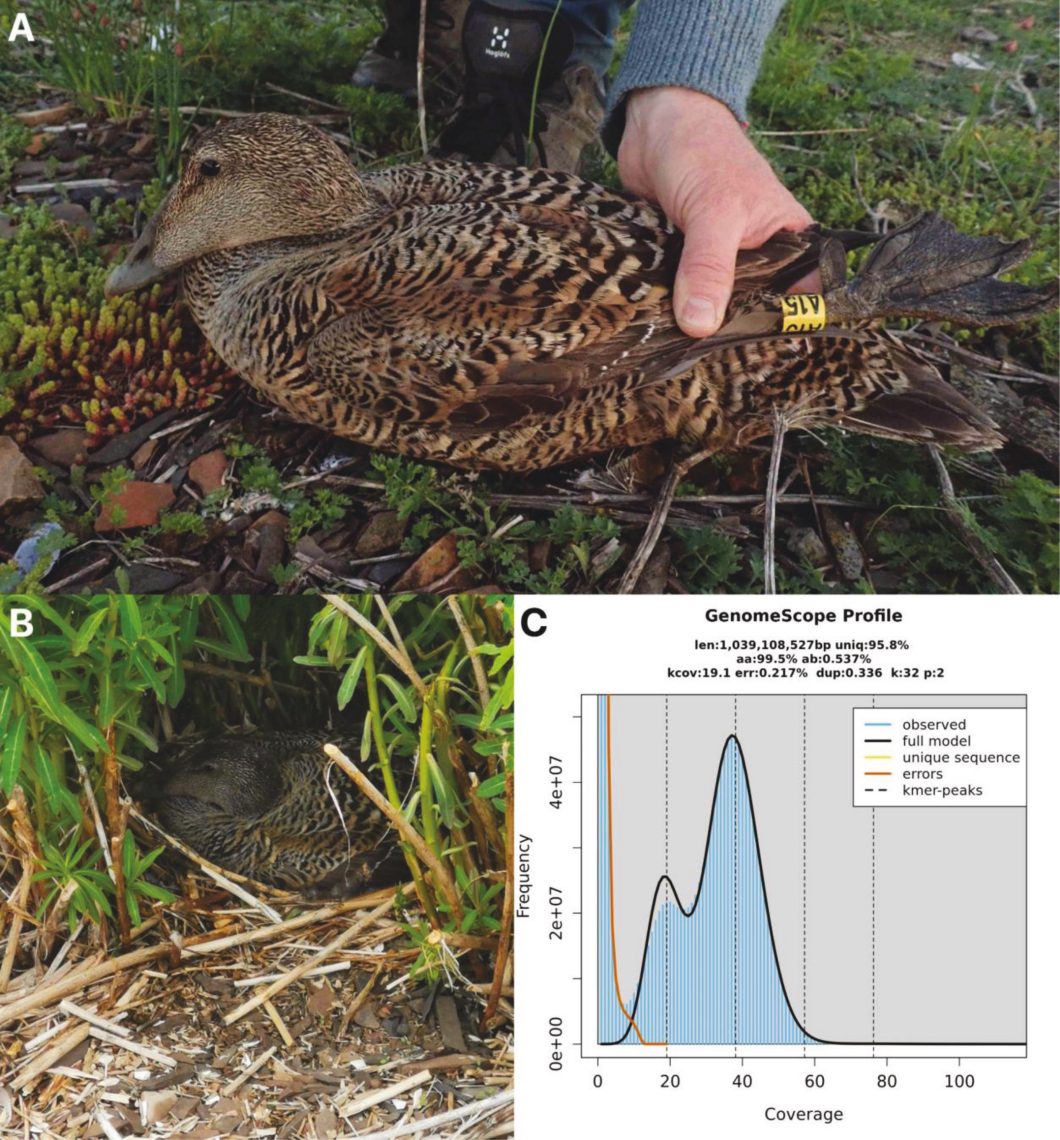
Sequenced specimen and genome profile. A) Photograph of the common eider (*Somateria mollissima*) bSomMol1 specimen used for genome sequencing. Also shown are the nesting habitat (b) and the GenomeScope profile (b) of the HiFi reads from the sequenced individual. This analysis estimates a 1,039 Mbp genome, with 0.537 % heterozygosity and bimodal pattern characteristic of a diploid genome. The left-hand peak of k-mers corresponds to k-mers from heterozygous regions of the genome, while the right-hand peak is from homozygous regions.

A blood sample from a female *S. mollissima* specimen (bSomMol1) was collected from Søndre Skjælholmen, Nesodden municipality, Viken county, Norway (59.852333°N, 10.724667°E), on 20 May 2021 (Figure 1a, b). The specimen was caught in a nest with three eggs and observed incubating again a short time after sampling. She was ringed with metal ring Stavanger Museum CA46355 and coloring ring yellow A15. The bird had a wing length of 303 mm and a body mass of 1,770 g. A blood sample was taken from the vein at the tarsus and preserved in ethanol, and stored in a thermos flask with water and ice until freezing at −20 °C in the laboratory.

The area where the eider duck was sampled is not a protected nature reserve, and MHs ringing license number 560 issued by the Norwegian Environment Agency and the blood sample was taken under permission FOTS 23171 from the Norwegian animal ethics authority Mattilsynet.

DNA isolation for PacBio long-read sequencing was performed using Circulomics Nanobind CBB BIG DNA kit and protocol according to manufacturer’s recommendations, including treatment with EtOH removal buffer (Circulomics, now PacBio company). Quality check of amount, purity, and integrity of isolated DNA was performed using Qubit BR DNA quantification assay kit (Thermo Fisher), Nanodrop (Thermo Fisher), and Fragment Analyzer (DNA HS 50kb large fragment kit, Agilent Tech.).

### Library preparation and sequencing for de novo assembly

Before PacBio HiFi library preparation, DNA was purified an additional time using AMPure PB beads (1:1 ratio). Approximately 7.5 µg of purified HMW DNA was sheared into an average fragment size of 15 to 20 kbp large fragments using the Megaruptor3 (Diagenode). For library preparation, 5 µg of fragmented DNA was used after the PacBio protocol for HiFi library preparation using the SMRTbell express template kit 2.0. The final HiFi library was size-selected with an 11 kbp cut-off using a BluePippin (Sage Biosciences) and sequencing was performed by the Norwegian Sequencing Centre on the PacBio Sequel II instrument. The library was sequenced on two 8M SMRT cells using the Sequel II Binding kit 2.2 and Sequencing chemistry v2.0.

A Hi-C library was prepared using the Arima High Coverage Hic (HiC+) kit, following the manufacturer’s recommendations (document part number A160162v01) and starting with 25 to 50 µL blood in EtOH. Final library quality was assayed as above in addition to qPCR using the Kapa Library quantification kit for Illumina (Roche Inc.). The library was sequenced with other libraries on the Illumina NovaSeq SP flowcell with 2 * 150 bp paired end mode at the Norwegian Sequencing Centre.

### Genome assembly and curation, annotation, and evaluation

A full list of relevant software tools and versions is presented in Table 1. KMC (Kokot et al. 2017) was used to count k-mers of size 32 in the PacBio HiFi reads, excluding k-mers occurring more than 10,000 times. GenomeScope (Ranallo-Benavidez et al. 2020) was run on the k-mer histogram output from KMC to estimate genome size, heterozygosity, and repetitiveness while ploidy level was calculated using Smudgeplot (Ranallo-Benavidez et al. 2020). HiFiAdapterFilt (Sim et al. 2022) was applied on the HiFi reads to remove possible remnant PacBio adapter sequences. The filtered HiFi reads were assembled using hifiasm (Cheng et al. 2021) with Hi-C integration resulting in a pair of haplotype-resolved assemblies, pseudo-haplotype one (hap1) and pseudo-haplotype two (hap2). Unique k-mers in each assembly/pseudo-haplotype were identified using meryl (Rhie et al. 2020) and used to create two sets of Hi-C reads, one without any k-mers occurring uniquely in hap1 and the other without k-mers occurring uniquely in hap2. k-mer filtered Hi-C reads were aligned to each scaffolded assembly using BWA-MEM (Li 2013) with −5SPM options. The alignments were sorted based on name using samtools (Li et al. 2009) before applying samtools fixmate to remove unmapped reads and secondary alignments and to add mate score, and samtools markdup to remove duplicates. The resulting BAM files were used to scaffold the two assemblies using YaHS (Zhou et al. 2022) with default options. FCS-GX (Astashyn et al. 2024) was used to search for contamination. Contaminated sequences were removed. If a contaminant was detected at the start or end of a sequence, the sequence was trimmed using a combination of samtools faidx, bedtools (Quinlan and Hall 2010) complement and bedtools getfasta. If the contaminant was internal, it was masked using bedtools maskfasta. The mitochondrion was searched for in contigs and reads using MitoHiFi (Uliano-Silva et al. 2023). Merqury (Rhie et al. 2020) was used to assess the completeness and quality of the genome assemblies by comparing to the k-mer content of the Hi-C reads. BUSCO (Manni et al. 2021) was used to assess the completeness of the genome assemblies by comparing against the expected gene content in the aves lineage. Gfastats (Formenti et al. 2022a) was used to output different assembly statistics of the assemblies. The assemblies were manually curated using PretextView and Rapid curation 2.0. Chromosomes (including sex chromosomes) were identified by mapping to chicken (GCF_016699485.2) and zebra finch (GCF_003957565.2), in addition to inspecting the Hi-C contact map in PretextView. BlobToolKit and BlobTools2 (Laetsch and Blaxter 2017), in addition to blobtk were used to visualize assembly statistics. To generate the Hi-C contact map, the Hi-C reads were mapped to the assemblies using BWA-MEM (Li 2013) using the same approach as above, before PretextMap was used to create a contact map which was visualized using PretextSnapshot.

**Table 1.** Software tools: versions and sources

Software tool	Version	Source
BlobToolKit	4.1.7	https://github.com/blobtoolkit/blobtoolkit
blobtk	0.5.1	https://github.com/blobtoolkit/blobtk
BUSCO	v5.4.7	https://gitlab.com/ezlab/busco
hifiasm	0.16.1-r375	https://github.com/chhylp123/hifiasm
KMC	v3.1.2rc1	https://github.com/refresh-bio/KMC
GenomeScope	v2.0	https://github.com/tbenavi1/genomescope2.0
HiFiAdapterFilt	v2.0.0	https://github.com/sheinasim/HiFiAdapterFilt
PretextView	0.2.5	https://github.com/wtsi-hpag/PretextView
PretextMap	0.1.9	https://github.com/wtsi-hpag/PretextMap
PretextSnapshot		https://github.com/wtsi-hpag/PretextSnapshot
meryl	1.3.0	https://github.com/marbl/meryl
BWA-MEM	v0.7.17	https://github.com/lh3/bwa
samtools	1.17	https://github.com/samtools/samtools
YaHS	yahs-1.1.91eebc2	https://github.com/c-zhou/yahs
FCS-GX	0.3.0	https://github.com/ncbi/fcs
Merqury	v1.3	https://github.com/marbl/merqury
AGAT	v1.0	https://github.com/NBISweden/AGAT
MitoHiFi	v2.2	https://github.com/marcelauliano/MitoHiFi
miniprot	0.11-r234	https://github.com/lh3/miniprot
GALBA	1.0.6	https://github.com/Gaius-Augustus/GALBA
RED	v2018.09.10	http://toolsmith.ens.utulsa.edu/
Funannotate	v1.8.13	https://github.com/nextgenusfs/funannotate
EvidenceModeler	v1.1.1	https://github.com/EVidenceModeler/EVidenceModeler
DIAMOND	v2.0.15	https://github.com/bbuchfink/diamond
InterProScan	v5.47-82	https://www.ebi.ac.uk/interpro/search/sequence/
EMBLmyGFF3	v2.2	https://github.com/NBISweden/EMBLmyGFF3
Earl Grey	v4.1.1	https://github.com/TobyBaril/EarlGrey
Flagger	v0.3.2	https://github.com/mobinasri/flagger
winnowmap	2.03	https://github.com/marbl/Winnowmap
Secphase	v0.4.3	https://github.com/mobinasri/secphase
DeepVariant	1.4.0	https://github.com/google/deepvariant
MUMmer	v4.0.0rc1	https://github.com/mummer4/mummer
Rapid curation 2.0	964d17e997e00c69f25940cf96d3658bda631147	https://github.com/Nadolina/Rapid-curation-2.0,
EMBOSS	6.6.0	https://emboss.sourceforge.net/

We annotated the genome assemblies using a pre-release version of the EBP-Nor genome annotation pipeline (https://github.com/ebp-nor/GenomeAnnotation). First, AGAT (https://zenodo.org/record/7255559) agat_sp_keep_longest_isoform.pl and agat_sp_extract_sequences.pl were used on the GRCg7b (GCA_016699485.1) chicken genome assembly and annotation to generate one protein (the longest isoform) per gene. Miniprot (Li 2023) was used to align the proteins to the curated assemblies. UniProtKB/Swiss-Prot (The UniProt Consortium et al. 2022) release 2022_03 in addition to the vertebrata part of OrthoDB v11 (Kuznetsov et al. 2022) were also aligned separately to the assemblies. Red (Girgis 2015) was run via redmask (https://github.com/nextgenusfs/redmask) on the assemblies to mask repetitive areas. In addition, we ran Earl Grey (Baril et al. 2024) to annotate transposable elements. GALBA (Stanke et al. 2006; Buchfink et al. 2015; Hoff and Stanke, 2018; Brůna et al. 2023; Li 2023) was run with the chicken proteins using the miniprot mode on the masked assemblies. The funannotate-runEVM.py script from Funannotate (https://zenodo.org/records/4054262) was used to run EvidenceModeler (Haas et al. 2008) on the alignments of chicken proteins, UniProtKB/Swiss-Prot proteins, vertebrata proteins and the predicted genes from GALBA. The resulting predicted proteins were compared with the protein repeats that Funannotate distributes using DIAMOND blastp and the predicted genes were filtered based on this comparison using AGAT. The filtered proteins were compared with the UniProtKB/Swiss-Prot release 2022_03 using DIAMOND (Buchfink et al. 2015) blastp to find gene names and InterProScan was used to discover functional domains. AGATs agat_sp_manage_functional_annotation.pl was used to attach the gene names and functional annotations to the predicted genes. EMBLmyGFF3 (Norling et al. 2018) was used to combine the fasta files and GFF3 files into a EMBL format for submission to ENA.

To evaluate the diploid assembly, we ran Flagger (Liao et al. 2023) to detect possible mis-assemblies. The HiFi reads were mapped to the diploid assembly (created by concatenating the two pseudo-haplotype) using winnowmap (Jain et al. 2022). Secphase (Liao et al. 2023) was run on the BAM file produced by winnowmap to correct the alignments of the reads by scoring them based on marker consistency, and selecting the alignment with the highest score as primary. SNPs were called from the corrected BAM file by DeepVariant (Poplin et al. 2018) using default parameters for PacBio HiFi data, and filtered to keep only biallelic SNPs. Flagger (Liao et al. 2023) was then run on the corrected BAM file together with the filtered VCF and categorized the diploid assembly into erroneous, duplicated, haploid, collapsed, and unknown regions.

To characterize the differences between the two pseudo-haplotypes, we ran nucmer from the MUMmer (Marçais et al. 2018) genome alignment system on the homologous chromosomes from the two pseudo-haplotypes. The resulting alignment was processed with dnadiff, also from MUMmer, producing a report listing the number of insertions, SNPs and indels between the two pseudo-haplotypes. EMBOSS (Rice et al. 2000) infoseq was used to calculate GC content of the different sequences.

## Results

### De novo genome assembly and annotation

**Fig. 2.**
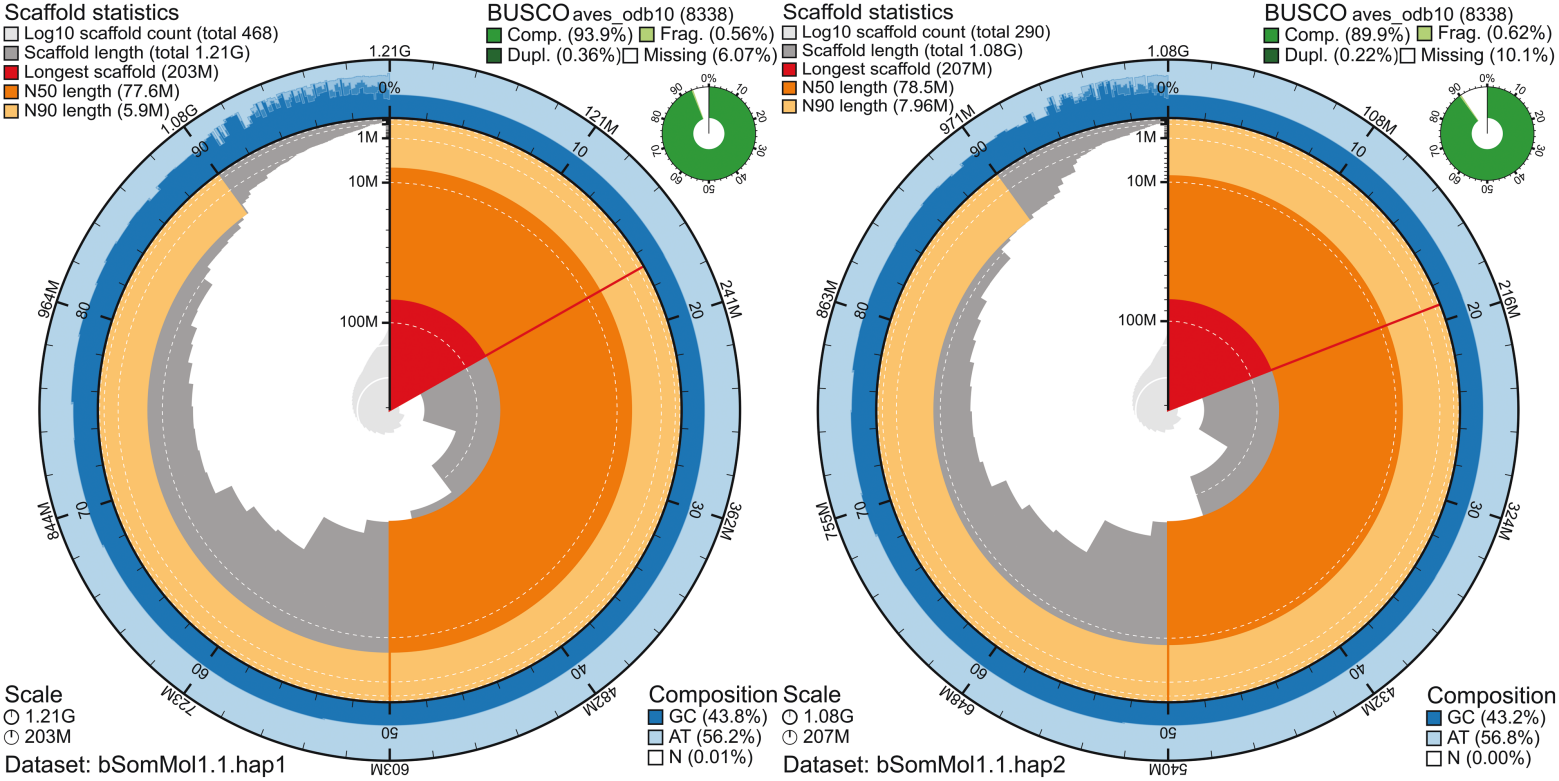
Metrics of the genome assemblies of *Somateria mollissima* bSomMol1.1 hap1 and hap2. The BlobToolKit Snailplots show N50 metrics and BUSCO gene completeness. The two outermost bands of the circle signify GC versus AT composition at 0.1% intervals. Third outermost shows the N90 scaffold length, while the fourth is N50 scaffold length. The line at almost a quarter of the circle, shows the size of the largest scaffold. All the scaffolds are arranged in a clockwise manner from the largest to the smallest and are shown in darker gray with white lines at different orders of magnitude, while the light gray shows cumulative count of scaffolds.

The genome from the female adult common eider (Figure 1), had an estimated genome size of 1.04 Gbp, with 0.54% heterozygosity and a bimodal distribution based on the k-mer spectrum (Figure 1c). A total of 39-fold coverage in Pacific Biosciences single-molecule HiFi long reads and 56-fold coverage in Arima Hi-C reads resulted in two haplotype-separated assemblies. The final assemblies have total lengths of 1,205 and 1,080 Mbp (Table 2 and Figure 2), respectively. Both of these are slightly larger than the k-mer based estimation, a difference that might be due to k-mers occurring more than 10,000 times are being excluded. hap1 and hap2 have scaffold N50 size of 77.5 and 78.5 Mbp, respectively, and contig N50 of 7.8 and 9.2 Mbp, respectively (Table 2, Figure 2 and Supplementary Table 1). Thirty-two automosomes were identified in both pseudo-haplotypes (numbered by length in hap1 with the homolog in hap2 receiving the same number) and the Z and W chromosomes were added to hap1.

**Table 2.** Genome data for *Someteria mollissima*, bSomMol1.

Project accession data			
Species		*Someteria mollissima mollissima*	
Specimen		bSomMol1	
NCBI taxonomy ID		76058	
BioProject		PRJEB65713	
BioSample ID		SAMEA112864454	
Isolate information		Female, blood	
**Raw data accessions**			
PacBio HiFi reads		ERX10619619-ERX10619620	2 PACBIO_SMRT (Sequel II) runs: 2.9 M reads, 44.1 Gbp
Hi-C Illumina reads		ERX10619531	1 ILLUMINA (Illumina NovaSeq S4) run: 193 M pairs of reads, 58.2 Gbp
**Genome assembly metrics**			
HiFi read coverage		39×	
Assembly accession		PRJEB61097	PRJEB62037
Assembly identifier		bSomMol1.hap1.1	bSomMol1.hap2.1
Span (Mbp)		1,205	1,080
Number of contigs		740	499
Contig N50 length (Mbp)		7.8	9.2
Longest contig (Mbp)		39.5	37.5
Number of gaps		272	209
Number of scaffolds		468	290
Scaffold N50 length (Mbp)		77.5	78.5
Longest scaffold (Mbp)		203.4	206.6
Consensus quality (QV) compared with Hi-C (compared with HiFi)		39.6052 (62.0327)	40.2342 (62.7658)
Both assemblies		39.891 (62.3637)	
*k*-mer completeness (percentage; compared with HiFi)		91.4473 (93.0159)	86.2129 (86.8272)
Both assemblies		94.9898 (97.1762)	
BUSCO*		C: 94.0% [S: 93.6%, D: 0.4%], F: 0.6%, M: 5.4%, *n*: 8,338	C: 89.9% [S: 89.7%, D: 0.2%], F: 0.6%, M: 9.5%, *n*: 8,338
Percentage of assembly mapped to chromosomes		91.13	93.18
Flagger^a^		H: 96.54%, D: 3.23%, E: 0.26%, C: 0.06%, U: 0.0%	H: 97.16%, D: 2.57%, E: 0.18%, C: 0.09%, U: 0.0%
mummer	Aligned bases	994,650,955 (99.0379%)	993,718,509 (98.8053%)
	Insertions (sum in bp)	10,541 (27,769,806)	8,612 (28,911,361)
	SNPs	2,369,345	2,369,345
	Indels	1,835,216	1,835,216
Sex chromosomes		ZW	
Organelles		(not identified)	(not identified)
**Genome annotation**			
Number of protein-coding genes		17,479	16,315
BUSCO*		C: 93.7% [S: 93.3%, D: 0.4%], F: 0.4%, M: 5.9%, *n*: 8,338	C: 89.9% [S: 89.6%, D: 0.3%], F: 0.4%, M: 9.7%, *n*: 8,338

^a^Flagger scores H = haploid, D = duplicated, E = error, C = collapsed, U = unknown.

^b^BUSCO scores based on the aves BUSCO set using v5.4.7. C = complete [S = single copy, D = duplicated], F = fragmented, M = missing, *n* = number of orthologues in comparison.

**Fig. 3.**
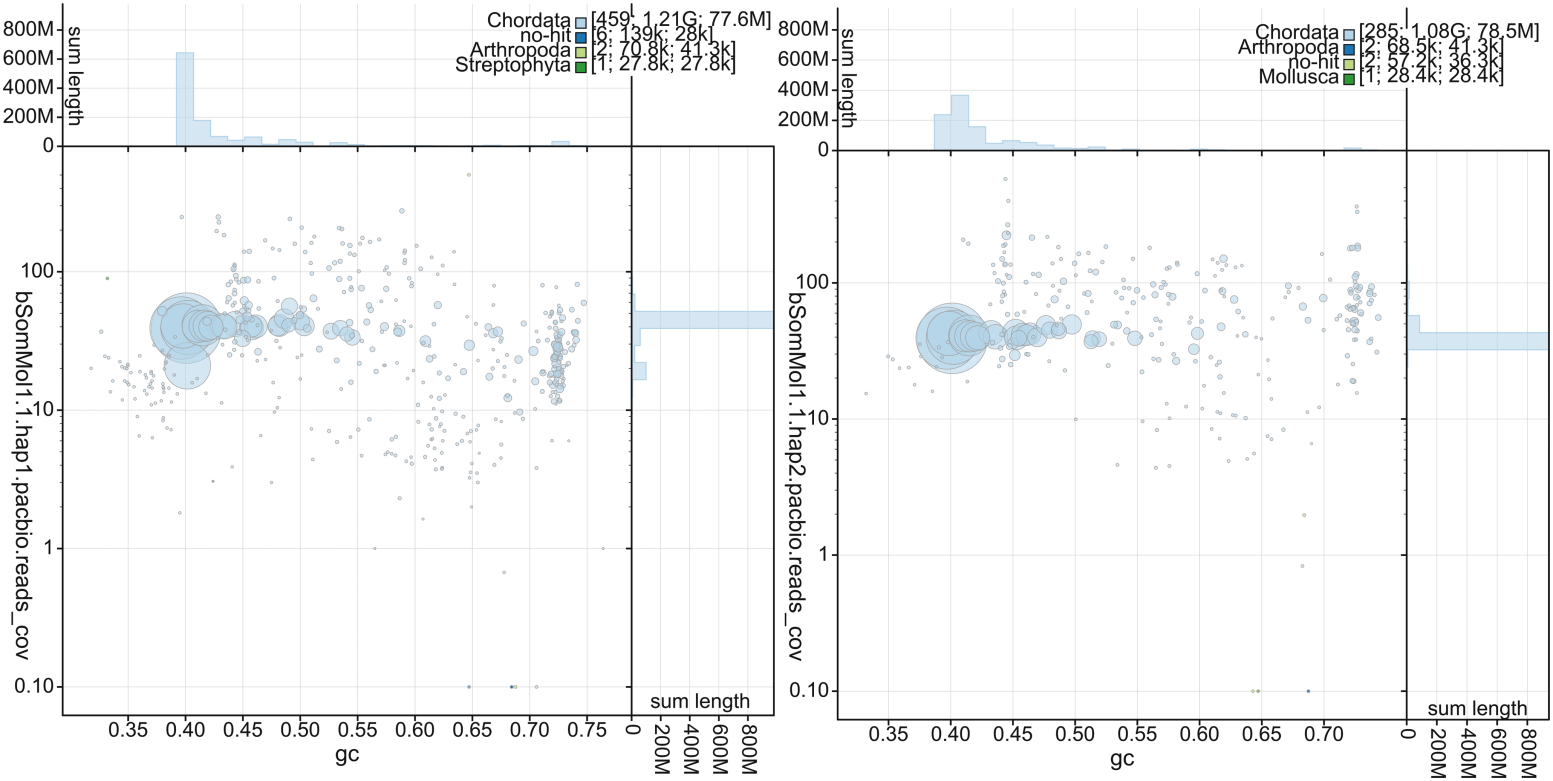
BlobToolKit GC-coverage plots of genome assemblies of *Somateria mollissima,* bSomMol1.1 hap1 and hap2. The scaffolds are marked by phylum. The size of the circles is in proportion to the length of the scaffolds. Histograms show the distribution of scaffold length sum along each axis.

**Fig. 4.**
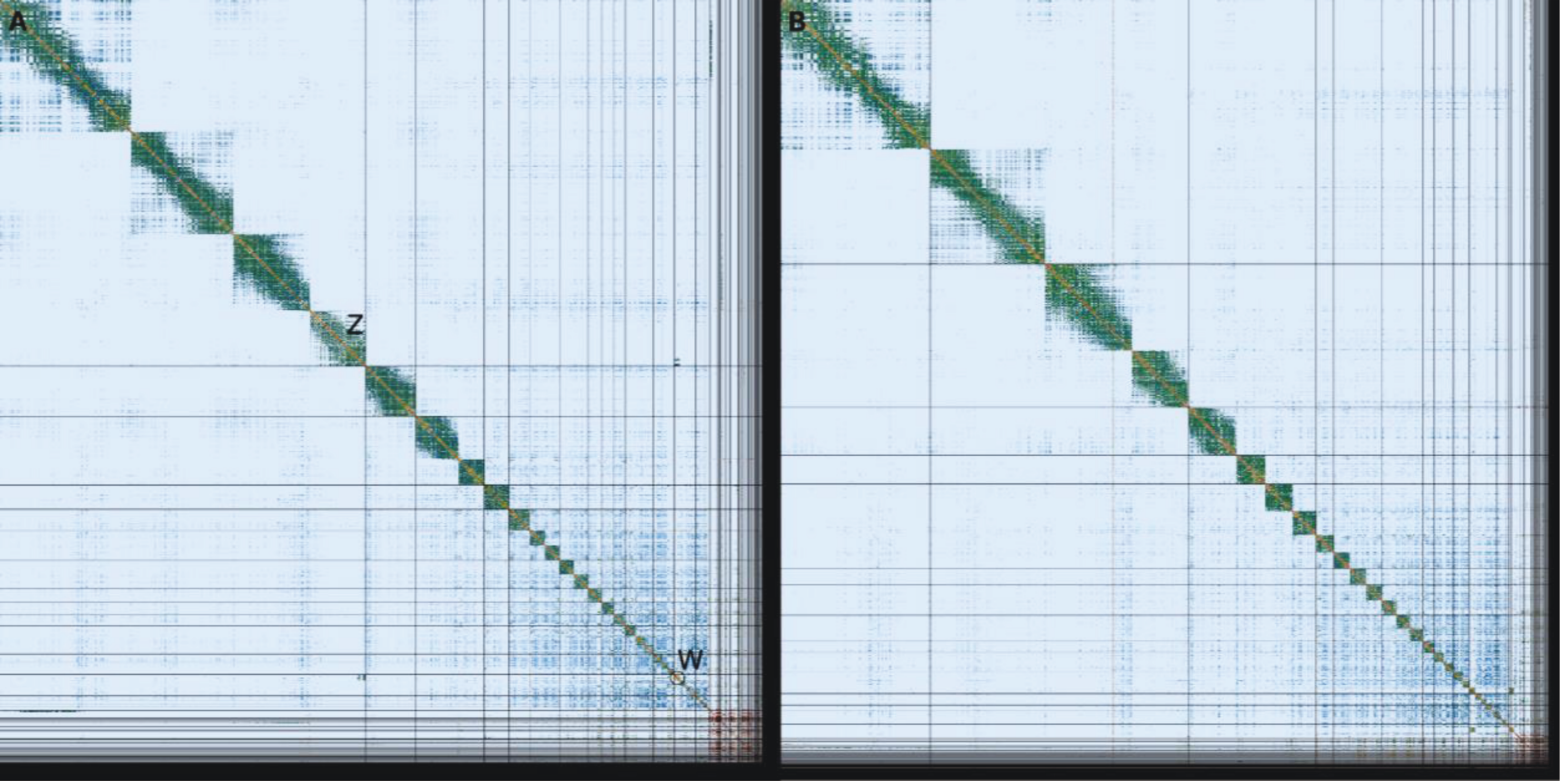
Hi-C contact map of genome assemblies of *Somateria mollissima* bSomMol1.1 hap1 and hap2. Both assemblies are visualized using PreTextSnapshot. a) hap1, b) hap2. Chromosomes are shown in order of size from left to right and top to bottom. Z and W are marked in hap1.

hap1 had 94.0% and hap2 89.9% complete BUSCO genes using the aves lineage set. When compared with a k-mer database of the Hi-C reads hap1 had a k-mer completeness of 91.4%, hap2 of 86.2%, and combined they have a completeness of 95.0%. Further, hap1 has an assembly consensus quality value (QV) of 39.6 and hap2 of 40.2, where a QV of 40 corresponds to one error every 10,000 bp, or 99.99% accuracy compared with a k-mer database of the Hi-C reads (QV 62.0 and 62.8, respectively, compared with a k-mer database of the HiFi reads). The GC content of the four largest autosomal chromosomes plus Z is similar (average around 40%, Figure 3 and Supplementary Table 1). The next four have around 41% to 42% GC content, but the smallest chromosomes have even higher GC content. Unplaced sequences (which include repetitive sequences) have the highest GC content, up to 60% (Figure 3 and Supplementary Table 1). The GC content is plotted against the coverage in Figure 3 showing that the largest chromosomes have similar GC content, while smaller ones are shifted toward higher GC content. The Hi-C contact map for the assemblies are shown in Figure 4, and show clear separation of the different chromosomes.

Flagger identified 96.53% of hap1 as haploid, 3.23% as duplicated, 0.26% as error regions, and 0.06% as collapsed. The respective percentages for hap2 are 97.16% haploid, 2.57% duplicated, 0.18% error, and 0.09% collapsed (Table 1). In a separate analysis using nucmer to align the two pseudo-haplotypes, 10,541 insertions were found in hap1 compared with hap2, 8,612 insertions in hap2 compared with hap1 and 2,369,345 SNPs and 1,835,216 indels between them. A total of 17,479 and 16,315 protein-coding genes were annotated in hap1 and hap2, respectively. In hap1, 17.84% of the genome assembly was annotated as a repetitive sequence, while in hap2, 14.62% (Supplementary Table 2).

## Discussion

The presented genome sequence of common eider increases the number of species reaching the EBP standards for genome assemblies (Lawniczak et al. 2022) within waterfowls (Anseriformes) to nine. High quality genome assemblies are an extremely valuable resource for investigating population structure, gene flow, and comparative and evolutionary genomics including synteny, evolutions of gene families and repeated elements (Tørresen et al. 2019; Formenti et al. 2022b; Lewin et al. 2022; Theissinger et al. 2023).

Assessing the quality of a genome assembly is not straight-forward. There are a multitude of methods available such as FRCurve (Vezzi et al. 2012) for comparing the characteristics of sequencing reads to the genome assembly, and QUAST (Mikheenko et al. 2018) for comparison to a known reference. In this study, we have utilized several well-established methods such as BUSCO (Manni et al. 2021) and Merqury (Rhie et al. 2020). BUSCO identifies genes that are expected to occur in the taxa under investigation, and gives a score where a higher percentage of genes found represents a more complete assembly. Here, 94.0% complete BUSCO genes (7,832 of the 8,338 Aves genes) are found in hap1 and 89.9% found in hap2 (7,497/8,338 genes). The difference is mainly due to the Z chromosome harboring 365 complete genes (4.4% of 8,338 genes). Only four BUSCO genes are found on the W chromosome, and these are all duplicated with duplicates on Z and autosomes. This corresponds well with other high-quality bird genome assemblies such as California scrub jay with 97.0% and 93.3% complete BUSCO genes in primary and alternate assemblies, respectively (DeRaad et al. 2023) and in black rail with 96.8% and 85.1% in primary and alternate assemblies, respectively (Hall et al. 2023).

With Merqury (Rhie et al. 2020), k-mers in the assemblies are compared with the k-mers in the reads, and assuming most k-mers would be incorporated in one or both assemblies, a higher concordance between the k-mers in the reads and the k-mers in the assemblies reflects a more complete assembly. Ideally, Merqury is run on a set of reads that has not been used to assemble the genome, but as an approximation we performed these analyses on both Hi-C and PacBio HiFi reads (Table 1). Although the HiFi reads are not independent because they were used to assemble the genome, this comparison likely represents a higher boundary of correctness and completeness. The Hi-C reads might contain junctions due to the Hi-C protocol (Lieberman-Aiden et al. 2009), thus having k-mers that do not actually occur in the genome. The numbers for this analysis would therefore represent a lower boundary of correctness and completeness.

Each pseudo-haplotype on its own is only 91.4% and 86.2% complete compared with k-mers from the Hi-C reads (93.0% and 86.8%, respectively, against the HiFi reads). With both pseudo-haplotypes combined we, however, achieve a 95% completeness (97% with HiFi), reflecting the value of resolving both haplotypes. This is similar to California scrub jay with a k-mer completeness of 92.4996 and 85.916 for primary and alternate assemblies, respectively, and 99.5694 completeness combined (DeRaad et al. 2023). In black rail, the e k-mer completeness is 92.65 and 78.18 for the primary and alternate assemblies, respectively, and 99.5403 for both combined (Hall et al. 2023).

Collapsed assemblies can switch between the two haplotypes, and might lead to wrong inferences in analyses, for instance by incorporating two variants that do not occur in nature in the nucleotide sequence. There are few methods that can be utilized to measure the correctness of (pseudo-)haplotype-resolved assemblies without access to sequencing data from the parents. One of the few is Flagger (Liao et al. 2023), which was first used to measure the correctness with regards to haploid, duplication, collapse, and error sequence in genome assemblies that were used to create a pangenome for humans (Liao et al. 2023). It is difficult to evaluate the numbers reported here (96.5% and 97.2% haploid in hap1 and hap2, respectively; Table 1) because the only comparison so far are the 47 assemblies in (Liao et al. 2023). The assemblies reported there are all listed as 99% haploid, compared with 96.5% and 97.2% here. However, they were all assembled in trio mode, that is, the parents were also sequenced, enabling the assembler to completely phase the assemblies. Over time, we expect more studies to evaluate haplotype-resolved genome assemblies using methods such as those implemented in Flagger.

The karyotype for common eider is likely 2*n* = 80 (Hammar 1970). In this study we identify 32 pairs of autosomes plus the sex chromosomes, leaving seven pairs of microchromosomes unidentified. These were unidentified despite visual inspection of the Hi-C contact map made from aligning the Hi-C reads to both pseudo-haplotypes simultaneously, and comparisons with the chromosomes in chicken and zebra finch genome assemblies. An alternative approach is to name the longest scaffolds as chromosomes (DeRaad et al. 2023). However, in many cases the unplaced contigs can be larger than the real microchromosomes. In hap1 for the common eider, the largest unplaced contig is 3 Mbp, almost as large as chromosome 28 and larger than chromosomes 29, 30, and 31 (1.6, 1.2, and 0.9 Mbp respectively). A recent telomer-to-telomer genome assembly on chicken resolved all chromosomes (Huang et al. 2023), with the shortest at 2.5 Mbp. In an unpublished common eider genome assembly (GCA_030142145), the shortest chromosome is 18.6 kbp. The two assemblies presented here match one-to-one for all chromosomes but chromosome 31 to GCA_030142145. While GCA_030142145 has 41 chromosomes identified (one more than the expected karyotype), the shortest chromosomes are smaller than the chromosomes in our assemblies and 13 of them are shorter than the shortest chicken chromosome. Due to the lack of description it is not known how these chromosomes were identified. We have been more conservative in what we call chromosomes and have therefore not classified 40 pairs. However, the chromosomal sequences are present in the assemblies as unplaced sequences.

In light of the rapidly changing Arctic environment and declining European eider populations over the past decades, this new genomic resource will play an important role to disentangle taxonomy, spatial structure and conservation needs of the breathtakingly beautiful common eider.

## Supplementary material

Supplementary material is available at http://www.jhered.oxfordjournals.org/ Journal online.

esae042_suppl_Supplementary_Tables

## Data Availability

Data generated for this study are available under ENA BioProject PRJEB65713 for EBP-Nor. Raw PacBio sequencing data for the common eider (ENA BioSample: SAMEA112864454) are deposited in ENA under ERX10619619-ERX10619620, while Illumina Hi-C sequencing data is deposited in ENA under ERX10619531. Pseudo-haplotype one can be found in ENA at PRJEB61097, while hap2 is PRJEB62037. The gene and transposable element annotations are available at https://zenodo.org/records/11159637 (DOI:10.5281/zenodo.11159636).
