# Supplementary material for: A chromosome-level genome assembly of the common eider, Somateria mollissima (Linnaeus, 1758)
Source: J Hered. 2024 Sep 4;116(3):363–72. doi: 10.1093/jhered/esae042 (PMC12130440; doi:10.1093/jhered/esae042)
Supplement: esae042_suppl_Supplementary_Tables [file esae042_suppl_supplementary_tables.docx]

**Supplementary Material**

##### Supplementary Table 1: Chromosomal pseudomolecules in the genome assembly of *Somateria mollissima* bSomMol1, hap1 and hap2.

|  | Hap1 | | | Hap2 | | |
| --- | --- | --- | --- | --- | --- | --- |
| Chromosome | INSDC accession | Size (Mb) | GC% | INSDC accession | Size (Mb) | GC% |
| 1 | OX598302.1 | 203.40 | 40.08 | OX598690.1 | 206.61 | 40.12 |
| 2 | OX598303.1 | 157.94 | 39.61 | OX598691.1 | 158.76 | 39.70 |
| 3 | OX598304.1 | 118.24 | 40.20 | OX598692.1 | 119.60 | 40.22 |
| 4 | OX598306.1 | 77.55 | 39.78 | OX598693.1 | 78.49 | 39.88 |
| 5 | OX598307.1 | 66.32 | 41.48 | OX598694.1 | 66.23 | 41.47 |
| 6 | OX598308.1 | 39.52 | 41.11 | OX598695.1 | 39.93 | 41.17 |
| 7 | OX598309.1 | 37.67 | 41.68 | OX598696.1 | 37.48 | 41.57 |
| 8 | OX598310.1 | 32.57 | 41.94 | OX598697.1 | 32.31 | 41.94 |
| 9 | OX598311.1 | 23.40 | 44.32 | OX598698.1 | 24.18 | 45.22 |
| 10 | OX598312.1 | 22.69 | 43.25 | OX598699.1 | 22.66 | 43.25 |
| 11 | OX598313.1 | 22.10 | 43.41 | OX598700.1 | 22.25 | 43.60 |
| 12 | OX598314.1 | 21.70 | 42.22 | OX598701.1 | 21.57 | 42.17 |
| 13 | OX598315.1 | 21.04 | 45.76 | OX598702.1 | 20.72 | 45.28 |
| 14 | OX598316.1 | 19.04 | 46.12 | OX598703.1 | 18.72 | 45.79 |
| 15 | OX598317.1 | 17.18 | 48.17 | OX598704.1 | 17.38 | 46.38 |
| 16 | OX598318.1 | 16.32 | 45.73 | OX598705.1 | 15.81 | 46.09 |
| 17 | OX598319.1 | 13.81 | 48.05 | OX598706.1 | 13.18 | 46.96 |
| 18 | OX598320.1 | 13.43 | 50.36 | OX598707.1 | 13.13 | 47.65 |
| 19 | OX598321.1 | 13.41 | 48.60 | OX598708.1 | 13.07 | 49.73 |
| 20 | OX598322.1 | 9.94 | 50.04 | OX598709.1 | 8.89 | 47.96 |
| 21 | OX598323.1 | 8.66 | 49.06 | OX598710.1 | 8.03 | 51.40 |
| 22 | OX598325.1 | 8.51 | 52.69 | OX598711.1 | 7.96 | 48.69 |
| 23 | OX598326.1 | 7.95 | 54.53 | OX598712.1 | 6.99 | 51.93 |
| 24 | OX598327.1 | 7.78 | 53.48 | OX598713.1 | 6.86 | 54.80 |
| 25 | OX598328.1 | 6.55 | 54.07 | OX598714.1 | 6.46 | 51.28 |
| 26 | OX598329.1 | 5.90 | 48.99 | OX598715.1 | 5.82 | 48.65 |
| 27 | OX598330.1 | 3.62 | 58.62 | OX598716.1 | 4.47 | 59.82 |
| 28 | OX598331.1 | 3.34 | 60.90 | OX598717.1 | 3.26 | 59.53 |
| 29 | OX598332.1 | 1.59 | 57.34 | OX598718.1 | 1.51 | 61.73 |
| 30 | OX598333.1 | 1.16 | 61.99 | OX598719.1 | 1.34 | 62.78 |
| 31 | OX598334.1 | 1.05 | 51.15 | OX598720.1 | 1.17 | 48.35 |
| 32 | OX598335.1 | 0.90 | 54.30 | OX598721.1 | 0.88 | 54.15 |
| W | OX598324.1 | 8.65 | 44.98 | - |  |  |
| Z | OX598305.1 | 85.55 | 40.16 | - |  |  |
| unplaced |  | 106.92 | 61.75 |  | 73.53 | 57.82 |

#####

##### Supplementary Table 2: Repetitive sequences and their classification in the genome assembly of *Somateria mollissima* bSomMol1, hap1 and hap2.

|  | Hap1 | | | Hap2 | | |
| --- | --- | --- | --- | --- | --- | --- |
| Repeat classification | Number | Coverage (bp) | Percentage of assembly | Number | Coverage (bp) | Percentage of assembly |
| DNA | 4,623 | 835,934 | 0.1 | 4,939 | 852,436 | 0.01 |
| LINE | 165,141 | 6,613,1947 | 5.5 | 141,690 | 58,229,586 | 5.4 |
| LTR | 27,238 | 16,805,133 | 1.4 | 47,391 | 33,691,573 | 3.1 |
| Other (Simple Repeat, Microsatellite, RNA) | 617,531 | 128,727,297 | 10.7 | 553,234 | 57,557,682 | 5.3 |
| SINE | 4,509 | 605,602 | 0.05 | 4,098 | 463,117 | 0.01 |
| Unclassified | 33,669 | 10,253,238 | 0.09 | 24,751 | 8,580,396 | 0.8 |
| Sum | 852,711 | 223,359,151 | 17.84 | 776,103 | 159,374,790 | 14.62 |
